# Safety and accuracy of robot-assisted pedicle screw fixation: a randomized controlled trial

**DOI:** 10.1097/JS9.0000000000004498

**Published:** 2025-12-19

**Authors:** Yawen Jiang, Jikui Qian, Ling Zhang, Yan Yu, Liming Cheng, Jinwu Wang, Ying Yao, Shaobai Wang

**Affiliations:** a Key Laboratory of Exercise and Health Sciences of Ministry of Education, Shanghai University of Sport, Shanghai, China; b Department of Spine Surgery, Tongji Hospital, School of Medicine, Tongji University, Shanghai, China; c Key Laboratory of Spine and Spinal Cord Injury Repair and Regeneration, Ministry of Education, Department of Spine Surgery, Tongji Hospital, School of Medicine, Tongji University, Shanghai, China; d Shanghai Key Laboratory of Orthopedic Implant, Department of Orthopedics, Ninth People’s Hospital Affiliated to Shanghai Jiao Tong University School of Medicine, Shanghai, China; e Department of Operation, Tongji Hospital, School of Medicine, Tongji University, Shanghai, China

**Keywords:** navigation-guided system, pedicle screw, randomized controlled trial, robot-assisted surgery

## Abstract

**Background:**

Accurate pedicle screw placement is essential yet challenging in spinal surgery. This trial evaluated whether a robotic system with biplane fluoroscopic positioning could improve accuracy, efficiency, and safety compared to conventional fluoroscopy-guided techniques.

**Materials and methods:**

This multicenter, parallel-group, randomized trial was conducted at three hospitals. A total of 70 patients requiring pedicle screw fixation were randomly assigned (1:1) to either the robot-assisted group (RG) or the conventional fluoroscopy-guided group (CG). In the RG, a robotic navigation system assisted in planning and localizing the guide pin entry point and trajectory based on a biplane algorithm that integrated anteroposterior and lateral fluoroscopic images with optical tracking data to achieve real-time spatial registration. This biplane positioning technique was derived from the principles of the Dual Fluoroscopic Imaging System (DFIS). In the CG, guide pin placement was performed under conventional fluoroscopic guidance. The primary outcome was the deviation of guide pin placement. Secondary outcomes included the cumulative number of exposures, total radiation dose, operative time, average time to establish a single channel, and number of channel adjustments.

**Results:**

The mean deviation of guide pin placement in the RG was significantly smaller than that in the CG (0.86 ± 0.57 mm vs. 2.55 ± 1.88 mm, *P* < 0.001). The RG also had significantly fewer cumulative number of exposures (4.56 ± 1.70 vs. 13.38 ± 19.01, *P* = 0.003) and lower total radiation doses (7.35 ± 3.33 μSv vs. 30.28 ± 97.41 μSv, *P* = 0.024). The operative time was similar between the groups (245.03 ± 67.06 min vs. 215.18 ± 83.08 min, *P* = 0.083). The number of channel adjustments was significantly lower in the RG (0.06 ± 0.29 vs. 0.28 ± 0.90*, P* = 0.014). There were no significant differences in the incidence of adverse events between the two groups.

**Conclusions:**

The robotic system with biplane fluoroscopic positioning improved guide pin accuracy and reduced radiation exposure, indicating its potential to enhance precision and safety in spinal surgery.

## Introduction

Pedicle screw internal fixation is one of the most commonly used techniques in spinal surgery. It provides early stabilization, enhances the rate and speed of spinal fusion, and corrects spinal deformities[1]. Due to the proximity of the spinal canal and surrounding vital structures, accurate screw placement is critical to avoid serious complications such as nerve injury, bleeding, or even paralysis. In addition, anatomical variability in pedicle morphology and vertebral body size across patients necessitates the use of intraoperative imaging to ensure accurate screw placement[2]. In traditional surgery, pedicle screws are manually placed under fluoroscopic guidance, with the success rate heavily dependent on the surgeon’s technical skill and experience. However, this approach often results in increased radiation exposure for both the patient and the surgical team^[3,4]^.

**HIGHLIGHTS**

- Most existing robotic systems depend on 3D imaging, adding procedural complexity and imposing higher equipment demands.
- This study aimed to evaluate the accuracy, radiation exposure, procedural efficiency, and safety of a novel surgical navigation and positioning system that utilizes dual-plane fluoroscopic imaging instead of conventional 3D imaging, in comparison to conventional fluoroscopy-guided surgery.
- The robot-assisted group demonstrated significantly lower guide pin deviation, reduced radiation exposure, and fewer intraoperative adjustments.
- The robotic system enhances guide pin placement accuracy and reduces radiation exposure without increasing operative burden, showing potential to improve safety and efficiency in spine surgeries.

Recent advances in robotic-assisted surgery, visual navigation, and spatial registration technologies have significantly improved the accuracy, feasibility, and efficiency of spinal procedures^[5–8]^. For example, the TiRobot system uses 3D-3D registration with an O-arm for intraoperative 3D imaging and optical tracking of the patient and robotic arm to achieve precise screw placement^[9,10]^. The Mazor X system employs 2D-3D registration using intraoperative fluoroscopic images matched with preoperative CT scans to guide the surgeon in screw placement^[11–13]^. While both systems improve surgical precision, they rely on 3D imaging equipment, which can increase radiation exposure and make the procedure more complex.

A research team led by the Massachusetts Institute of Technology, Massachusetts General Hospital, and Harvard Medical School developed a dual fluoroscopic imaging system (DFIS), enabling dynamic 3D tracking and analysis of *in vivo* bone and joint motion^[14,15]^. This system has been applied to intraoperative joint surgical navigation[16] and *in vivo* kinematic analysis of bony structures in the lumbar spine^[17,18]^ . This method is considered the gold standard for motion analysis, with reported repeatability of less than 0.3 mm in translation and 0.7° in rotation for spinal 6-degree-of-freedom (6DOF) kinematics[19]. The biplane algorithm developed for this system is able to calculate the guide pin trajectory and robotic arm movement based on intraoperative anteroposterior and lateral fluoroscopic images[20]. This approach reduces the need for repeated intraoperative fluoroscopic imaging and eliminates the reliance on specialized 3D equipment such as 3D-CT, minimizing radiation exposure and reducing the need for additional hospital resources.

The all-in-one orthopedic robot (AIOOR) surgical navigation and positioning system (Droidsurg Medical Co., Ltd., Shanghai, China) combines robotic-assisted technology with biplane algorithmic positioning, offering a novel solution for pedicle screw internal fixation. Unlike existing robotic systems, such as the TiRobot and Mazor X, which require 3D imaging, the AIOOR system leverages dual fluoroscopic imaging, which is less complex, reduces radiation exposure, and lowers equipment demands. This study aims to evaluate the accuracy and safety of the AIOOR surgical navigation and positioning system in pedicle screw internal fixation, comparing it to conventional fluoroscopy-guided surgery. This study was conducted in accordance with the TITAN Guidelines 2025[21].

## Methods

### Study design

This was a multicenter, parallel-group, randomized controlled trial conducted at three hospitals in China. The study was approved by the respective institutional ethics committees, and written informed consent was obtained from all participants prior to enrollment. The trial was registered in the Chinese Clinical Trial Registry and was reported in accordance with the Consolidated Standards of Reporting Trials (CONSORT) guidelines[22].

### Participants

Eligible participants were adults scheduled to undergo open or minimally invasive fluoroscopy-guided pedicle screw fixation. Inclusion criteria were as follows: (1) age between 18 and 80 years; (2) no restriction on sex; and (3) provision of signed informed consent. Exclusion criteria were as follows: (1) known allergy to metal or multiple drugs; (2) pregnancy or lactation; (3) coagulation disorders; (4) prior failed spinal surgery at the same segment; (5) participation in other clinical trials involving drugs or devices within the past 3 months; (6) pedicle deformity; and (7) other conditions deemed by investigators to preclude participation.

### Randomization and blinding

Patients were randomized 1:1 to the robot-assisted group (RG) or the conventional fluoroscopy-guided group (CG) using a centralized computer-generated system. Patients and surgeons were not blinded due to the nature of the interventions. All surgeries in both groups were performed by the same experienced surgical team, ensuring consistency in skill level and minimizing variability due to surgeon experience. All outcomes were evaluated by an independent institute, and both assessors and statisticians were blinded to group allocation.

### Robot-assisted group

#### Robotic system

**Figure 1.**
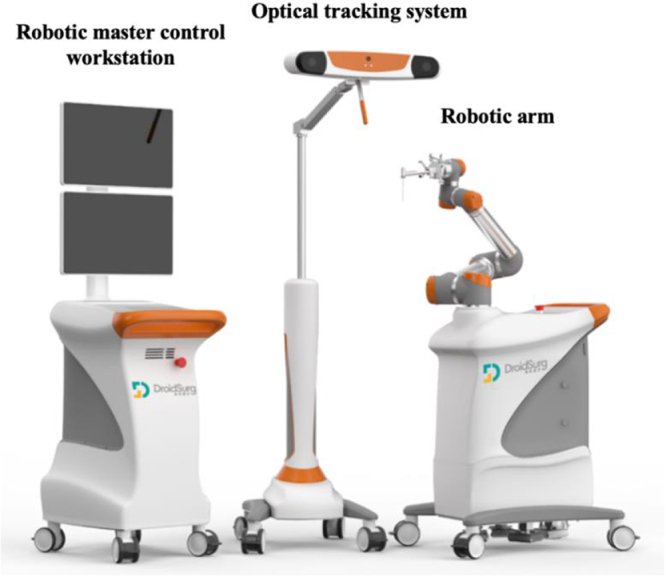
Main components of the AIOOR surgical navigation and positioning system, including the robotic arm, optical tracking system, and master control workstation.

The RG procedures utilized the AIOOR Surgical Navigation and Positioning System (Droidsurg Medical Co., Ltd., Shanghai, China), consisting of an optical tracking system, robotic arm, and master control workstation (Fig. 1). The optical tracking system employed a binocular camera to detect spatial positions of trackers attached to the robot and patient. The workstation performed image processing, trajectory planning, coordinate calculation, and robotic arm control. The robotic arm, with 6DOF, executed planned trajectories.

#### Surgical procedure

**Figure 2.**
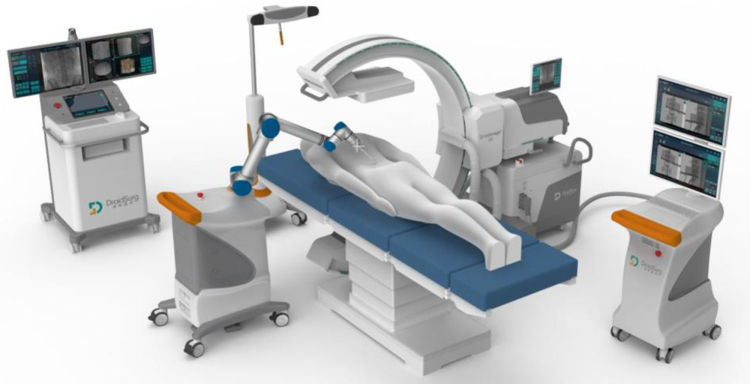
Integration of the AIOOR surgical navigation and positioning system with the C-arm fluoroscopy unit in the operating room.

**Figure 3.**
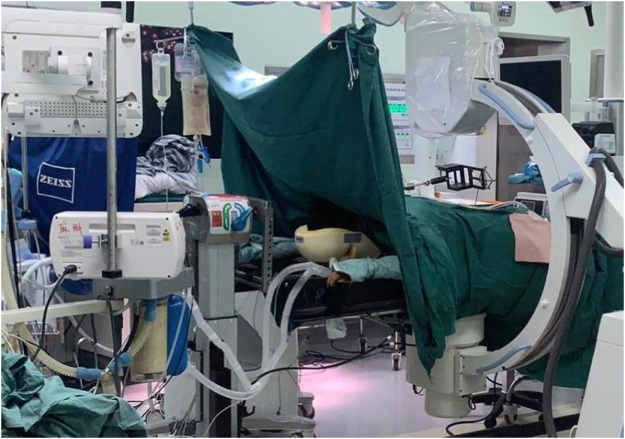
Intraoperative acquisition of anteroposterior and lateral fluoroscopic images using the C-arm for spatial registration.

**Figure 4.**
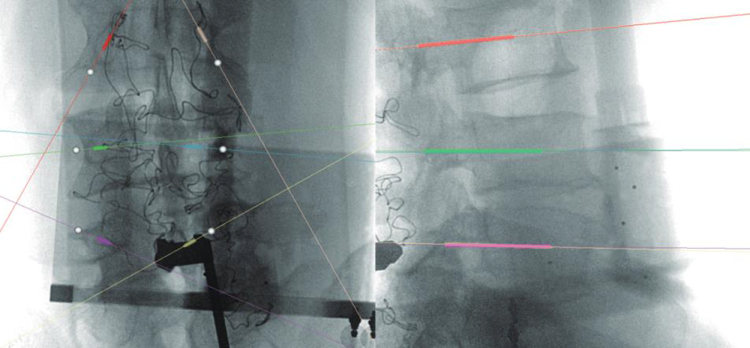
Surgical trajectory planning at the robotic master control workstation based on intraoperative fluoroscopic images.

**Figure 5.**
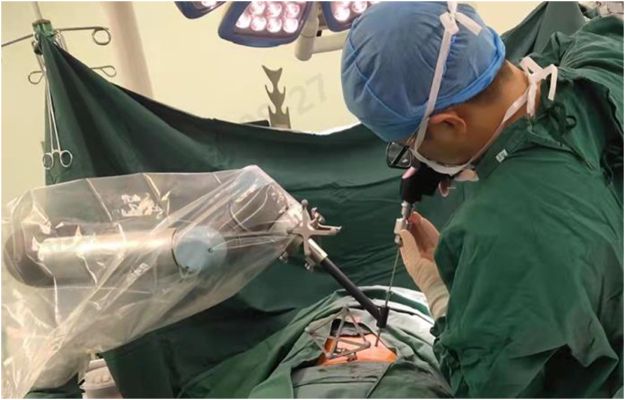
Robotic arm positioning at the planned entry point and guide pin placement through the cannula.

Patients were positioned prone under general anesthesia. The surgery was performed under sterile conditions. Calibration targets with reflective spheres were mounted on the robotic arm, the C-arm, and near the spinous processes of the vertebrae adjacent to the surgical site. An optical tracking system was used to collect spatial data from the calibration targets (Fig. 2). Anteroposterior and lateral fluoroscopic images of the patient were acquired using the C-arm (Fig. 3). The spatial data and fluoroscopic images were imported into the robotic master control workstation, and the spatial registration was achieved using a registration algorithm that aligned the image coordinate system with the robotic coordinate system, thereby enabling accurate robotic arm navigation. This dual-plane spatial positioning method was derived from the principles of the DFIS, allowing for real-time intraoperative localization without requiring 3D imaging. The surgeon planned the surgical channel at the robotic master control workstation (Fig. 4). The robotic arm automatically moved along the planned trajectory to the target position, from which the entry point, trajectory, and depth for guide pin placement were determined. The guide pin was placed through a cannula (Fig. 5), and its position was confirmed using both fluoroscopy and the robotic navigation system. Finally, the pedicle screw was inserted either percutaneously or through an open approach, depending on the clinical requirements.

### Conventional fluoroscopy-guided group

In the CG, guide pins were placed under fluoroscopic guidance based on the surgeon’s experience. After identifying the pedicle, a skin incision was made, a soft tissue channel was created, and the guide pin was inserted into the pedicle. Fluoroscopic imaging was used to confirm the accuracy of the guide pin placement, with additional imaging performed as necessary to ensure correct positioning.

### Outcomes and measurements

#### Primary outcome

**Figure 6.**
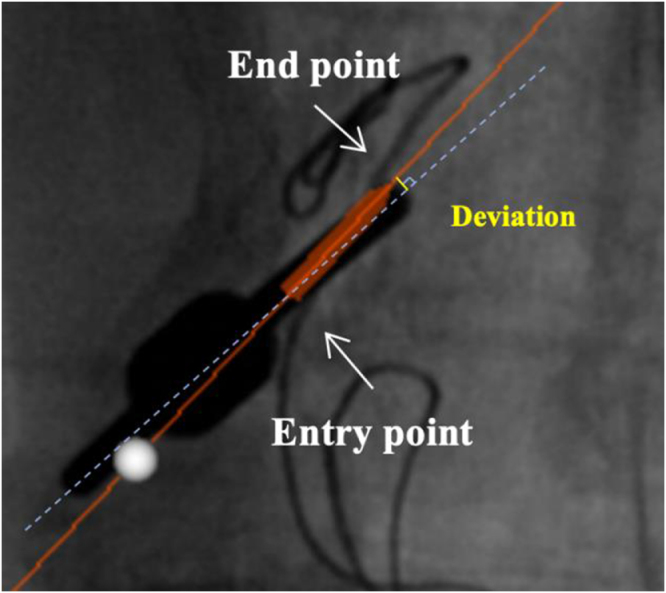
Measurement of guide pin placement deviation: perpendicular distance between the planned trajectory and the actual placement position at the entry and end points.

The primary outcome was the deviation of the guide pin, defined as the distance between the planned trajectory and the actual guide pin position as determined from intraoperative fluoroscopic images^[23,24]^, reflecting the accuracy of guide pin placement. To ensure objective assessment, deviations were measured postoperatively by independent evaluators blinded to group allocation and not involved in the procedures. Measurements were performed in the coronal and sagittal planes by calculating the perpendicular distance from the midline of the planned trajectory to the midline of the actual guide pin position (in mm). For each guide pin, deviations at the entry and end points were calculated as the square root of the summed squared deviations in both planes (Fig. 6).

$$Deviation=\;\sqrt{coronal\;\;deviation^{2}\;+sagittal\;\;deviation^{2}\;}$$

The deviation of the guide pin was calculated as the mean of the entry point and end point deviations.

#### Secondary outcomes

The secondary outcomes included intraoperative radiation exposure, procedural efficiency, and the number of channel adjustments. Specifically, the cumulative number of exposures and the total radiation dose (in μSv) were recorded for each patient from the initiation of intraoperative imaging to the completion of the final guide pin placement. Procedural efficiency was evaluated using the total operative time and the average time to establish a single channel (in minutes). Channel adjustments were defined as the number of times the initially planned trajectory required intraoperative modification or redirection during channel establishment. All secondary outcome data were assessed postoperatively by independent evaluators blinded to group allocation.

### Adverse events

All adverse events were recorded intraoperatively, on postoperative days 0–7, and at postoperative day 30 ± 7. The incidence and number of adverse events were documented. For serious adverse events, device-relatedness was assessed, and details of treatment and outcomes were recorded.

### Sample size calculation and statistical analysis

Sample size estimation was based on an assumed mean deviation of 2.60 mm in the CG and 1.92 mm in the RG, with a common standard deviation (SD) of 0.8 mm. With α = 0.05, 90% power, and 1:1 allocation, 31 patients per group were required (version 14.0, NCSS, Kaysville, UT, USA). Allowing for ~10% dropout, 68 patients were planned.

Continuous variables were presented as mean ± SD, and categorical variables as counts and percentages. The analysis was conducted according to the modified intention-to-treat (mITT) principle, whereby all randomized patients who underwent at least one guide pin placement were included. Patients who withdrew prior to surgery and had no outcome data were excluded from the analysis. The Shapiro–Wilk test was used to assess the normality of continuous variables. Between-group comparisons of continuous variables were performed using independent-samples *t*-tests for normally distributed data and the Mann–Whitney *U* test for non-normally distributed data. Chi-square tests were used for categorical variables with sufficient expected cell counts, while Fisher’s exact test was applied when expected frequencies were low. A sensitivity analysis using linear regression with preoperative diagnosis as a covariate was performed for the primary outcome. Two-sided *P* < 0.05 was considered significant. Statistical analyses were performed using SPSS version 26.0 (IBM Corp., Armonk, NY, USA).

## Results

**Figure 7.**
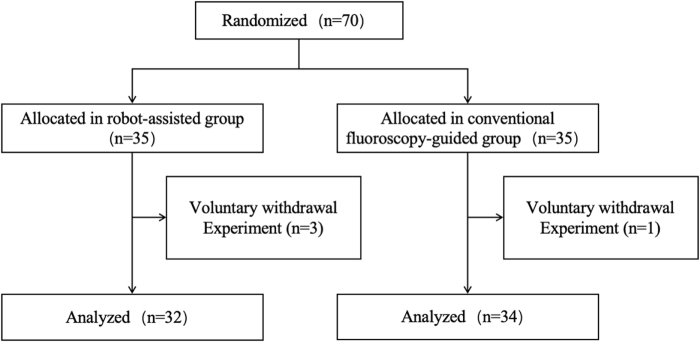
Study flowchart outlining patient randomization, inclusion, and final analysis.

A total of 70 patients were recruited for this study and randomized 1:1 into two groups: 35 patients in the RG and 35 in the CG. Patients underwent surgery between March 2021 and June 2023. Four patients (5.71%) withdrew and did not undergo surgery. According to the mITT principle, 66 patients (RG: 32, CG: 34) who underwent at least one guide pin placement were analyzed. The flowchart of the trial is shown in Fig. 7.

### Demographic and surgical characteristics

Patient characteristics are shown in Table 1. The mean age of patients at baseline was 61.41 ± 11.44 years, with 32 (48.48%) female patients. Degenerative disease was the most common diagnosis (RG: 81.3%; CG: 55.9%). In total, 344 guide pins were inserted (RG: 152; CG: 192), averaging 5.21 ± 1.64 per patient.

**Table 1:** Demographic data of the study cohort.

	Robot-assisted group (*N* = 32)	Conventional fluoroscopy-guided group (*N* = 34)	*P*-value
Sex (*n* %)			0.086
Male	13 (40.6)	21 (61.8)	
Female	19 (59.4)	13 (38.2)	
Age (years)	62.34 (11.14)	60.51 (11.99)	0.397
BMI (kg/m^2^)	24.76 (3.62)	25.10 (3.75)	0.697
Preoperative diagnosis(*n* %)			0.116
Degenerative disease	26 (81.3)	19 (55.9)	
Vertebral fracture	5 (15.6)	12 (35.3)	
Degenerative scoliosis	1 (3.1)	1 (2.9)	
Other	0 (0.0)	2 (5.9)	

### Primary outcomes

The deviation of the guide pin placement was significantly smaller in the RG compared to the CG (0.86 ± 0.57 mm vs. 2.55 ± 1.88 mm, *P* < 0.001) (Table 2). In the sensitivity analysis adjusting for preoperative diagnosis, patients in the CG had, on average, 1.80 mm greater deviation than those in the RG (95% CI 1.49–2.11, *P* < 0.001), confirming the robustness of the primary result.

**Table 2:** Primary outcome in the robot-assisted group and the conventional fluoroscopy-guided group.

	Robot-assisted group (*N* = 32)	Conventional fluoroscopy-guided group (*N* = 34)	Mean difference (95% CI)	*P*-value
Deviation of guide pin placement (mm)	0.86 (0.57)	2.55 (1.88)	−1.69 (−1.97 to −1.40)	<0.001

### Secondary outcomes

The RG required fewer cumulative number of exposures (4.56 ± 1.70 vs. 13.38 ± 19.01, *P* = 0.003) and lower radiation doses (7.35 ± 3.33 μSv vs. 30.28 ± 97.41 μSv, *P* = 0.024) compared with the CG. The average operative time was comparable between the groups (RG: 245.03 ± 67.06 min vs. CG: 215.18 ± 83.08 min, *P* = 0.083). Regarding procedural efficiency, the average time to establish a single channel was slightly shorter in the RG group, although the difference was not statistically significant (5.15 ± 2.51 min vs. 5.80 ± 3.95 min, *P* = 0.928). The RG required fewer channel adjustments (0.06 ± 0.29 vs. 0.28 ± 0.90, *P* = 0.014) (Table 3).

**Table 3:** Secondary outcomes in the robot-assisted group and the conventional fluoroscopy-guided group.

	Robot-assisted group (*N* = 32)	Conventional fluoroscopy-guided group (*N* = 34)	*P*-value
Cumulative number of exposures (*n*)	4.56 (1.70)	13.38 (19.01)	0.003
Total radiation dose (μSv)	7.35 (3.33)	30.28 (97.41)	0.024
Operative time (min)	245.03 (67.06)	215.18 (83.08)	0.083
Average time to establish a single channel (min)	5.15 (2.51)	5.80 (3.95)	0.928
Number of channel adjustments (*n*)	0.06 (0.29)	0.28 (0.90)	0.014

Operative time = Operative end time − Operative start time in minutes.

### Safety and adverse events

A total of 11 (34.4%) patients in the RG experienced 15 adverse events, while 10 (29.4%) patients in the CG experienced 13 adverse events. Most adverse events were mild to moderate in severity. One serious adverse event (COVID-19) occurred in the RG. All adverse events were considered unrelated to the robot-assisted procedure (Table 4). There were no statistically significant differences in the incidence of adverse events or serious adverse events between the RG and CG (*P* > 0.05). All events had a favorable clinical outcome.

**Table 4:** Summary of adverse events observed during the study.

	Robot-assisted group (*N* = 32), *n* (%)	Conventional fluoroscopy-guided group (*N* = 34), *n* (%)	Severity	*P*-value
Elevated liver enzymes	2 (6.2)	1 (2.9)	Mild	0.608
Vomiting	3 (9.4)	2 (5.9)	Mild	0.668
Dizziness	1 (3.1)	1 (2.9)	Mild	1.000
Wound pain	1 (3.1)	0 (0.0)	Mild	0.485
Fever	2 (6.2)	1 (2.9)	Mild	0.608
Pneumonia	1 (3.1)	2 (5.9)	Moderate	1.000
COVID-19 infection	1 (3.1)	0 (0.0)	Severe	0.485
Diarrhea	1 (3.1)	0 (0.0)	Mild	0.485
Upper respiratory infection	1 (3.1)	0 (0.0)	Mild	0.485
Musculoskeletal pain	1 (3.1)	0 (0.0)	Mild	0.485
Urethral pain	0 (0.0)	1 (2.9)	Mild	1.000
Angina	0 (0.0)	1 (2.9)	Mild	1.000
Hypokalemia	0 (0.0)	1 (2.9)	Mild	1.000
Hypoproteinemia	0 (0.0)	1 (2.9)	Moderate	1.000
Extremity venous thrombosis	0 (0.0)	1 (2.9)	Moderate	1.000
Vertigo	1 (3.1)	1 (2.9)	Mild	1.000

## Discussions

This multicenter, randomized, parallel-controlled trial evaluated the accuracy and safety of the AIOOR surgical navigation and positioning system for pedicle screw placement in spinal surgery. The results showed that the RG group had significantly smaller deviations in guide pin placement compared to the CG, indicating superior accuracy. Additionally, the RG group required fewer fluoroscopic exposures and experienced a lower total radiation dose, reducing radiation exposure for both patients and surgeons. While the operative time was similar between the RG and CG groups, the RG group required fewer channel adjustments, further demonstrating the efficiency of the AIOOR system in optimizing the surgical process without increasing intraoperative burden.

The deviation of guide pin placement using the AIOOR surgical navigation and positioning system in this study was 1.69 mm less compared to the conventional fluoroscopic guidance, indicating that the accuracy of placement using the surgical navigation and positioning system was significantly better than that of conventional fluoroscopic guidance. Our findings are consistent with those from other studies evaluating robotic systems for pedicle screw placement^[8,10,25]^. In a clinical study of the TiRobot system, the deviation of guide pin placement in the robot-assisted group (RG) was 1.47 mm[23]. Another study using the same system reported entry point and end point deviations of 1.88 and 1.70 mm, respectively[26]. In a controlled trial of the SpineAssist system, the screw placement deviation was 1.12 ± 0.41 mm[27], and another retrospective study recorded a deviation of 2.0 ± 1.2 mm[28]. In comparison, the AIOOR system in our study demonstrated a deviation of 0.86 ± 0.57 mm, which is lower than the data from the relevant literature and significantly better than conventional fluoroscopic guidance. The AIOOR system employs a dual-plane registration method that integrates anteroposterior and lateral fluoroscopic images with optical tracking to reconstruct 3D spatial relationships in real time. Prior validation studies of dual fluoroscopic imaging have demonstrated sub-millimeter translational accuracy and rotational errors below 1°, supporting its ability to achieve precision comparable to 3D-based navigation platforms while avoiding intraoperative CT or O-arm scans^[15,19]^. Clinically, even small deviations in pedicle screw placement can cause cortical breaches, neurovascular injury, or suboptimal screw fixation, potentially compromising spinal fusion and long-term stability. By reducing guide pin deviation, the AIOOR system may lower complication risk, enhance surgical safety, and improve patient outcomes.

In terms of radiation exposure, the cumulative number of exposures and the total radiation dose in the RG were significantly lower than those in the CG, which is consistent with previous studies showing reduced radiation exposure with robotic systems^[10,29–31]^. A meta-analysis reported that the cumulative exposure dose was 11.03–38.87 μSv for robotic-assisted procedures and 18.9–70.5 μSv for conventional manual procedures[32]. In our study, the cumulative number of exposures in the RG was 4.56 ± 1.70, and the total radiation dose was 7.35 ± 3.33 μSv, which is lower than that in other studies. This may be attributed to the fact that the AIOOR system in this study only required anteroposterior and lateral fluoroscopy, without the use of 3D-CT reconstruction like traditional robotic systems. The relatively large SD in the CG group (97.41 μSv) was mainly attributable to a few complex cases requiring repeated fluoroscopic confirmation. Overall, the AIOOR system may help reduce both the frequency of fluoroscopic exposures and the total radiation dose, thereby minimizing the radiation exposure for both patients and surgeons.

In our study, the average operative time was longer in the RG compared with the CG (245.03 ± 67.06 min vs. 215.18 ± 83.08 min, *P* = 0.083), reflecting a modest increase of about 30 min that did not reach statistical significance. Similar findings have been reported in previous studies, where robotic-assisted procedures often required additional time for system setup and registration[33]. The longer duration in our trial may be related to robotic system preparation, image registration, and trajectory planning[34]. However, several studies have demonstrated that operative time decreases significantly after the initial learning curve^[35,36]^, typically after 20–25 cases, as surgeons and operating teams become more familiar with the workflow[37]. From an economic perspective, although we did not evaluate cost-effectiveness, previous analyses suggest that robotic systems can offset their higher upfront costs in high-volume centers. For example, Menger *et al* estimated annual savings of approximately USD 600 000 by reducing revisions and length of stay[38]. In addition, our study found that the RG group required significantly fewer channel adjustments compared with the CG group, and the time to establish a single channel was slightly shorter. These findings suggest that the AIOOR system may enhance procedural accuracy and intraoperative efficiency. The reduced need for adjustments indicates more precise initial guide pin placement, which could help mitigate – though not fully offset – the additional setup time. Taken together, the AIOOR system demonstrated advantages in precision and reduced intraoperative corrections, with only a modest, non-significant increase in operative time, consistent with prior evidence that efficiency and economic value may improve as experience accumulates and adoption expands in high-volume centers.

From a clinical perspective, it is important to note that both the RG and CG groups were operated on by highly experienced surgeons from leading hospitals, who typically have extensive expertise in minimizing fluoroscopic exposure. Despite this, the AIOOR system still demonstrated significant advantages, particularly in reducing radiation exposure and improving accuracy. This indicates that even in the hands of experienced surgeons, robotic assistance can provide measurable improvements in safety and efficiency. Given these advantages, the system may be even more beneficial for less-experienced surgeons, for whom accurate guide pin placement and radiation control can be more challenging. Moreover, in high-volume spine surgery centers, where minimizing radiation and streamlining workflows are critical, broader adoption of this system could lead to further improvements in precision, efficiency, and patient safety.

This multicenter, randomized, parallel clinical trial has certain limitations. The sample size was modest, which may limit generalizability. The inclusion of different pathologies and surgical approaches, while reflecting real-world practice, may have introduced variability. The follow-up period was short, preventing evaluation of long-term outcomes. In addition, the influence of surgeon experience was not specifically analyzed.

Future studies should focus on several aspects. First, larger-scale, multicenter trials are warranted to validate the findings across diverse patient populations (e.g., age or comorbidity status) and surgical teams. Second, while the robotic system primarily assists in trajectory planning and guide pin placement, future studies will incorporate subgroup analyses based on pathology, surgical approach, and surgeon experience to further clarify its context-specific benefits. Third, extended follow-up is required to evaluate long-term clinical and radiographic outcomes, including screw stability, fusion rates, and delayed complications. Finally, cost-effectiveness analyses should be incorporated to assess the balance between improved accuracy, reduced radiation exposure, and the financial implications of implementing robotic-assisted technology in clinical practice.

## Conclusion

This multicenter, randomized, parallel-controlled trial demonstrated that the AIOOR surgical navigation and positioning system, utilizing biplane fluoroscopic positioning, significantly improved guide pin placement accuracy, reduced radiation exposure, and enhanced surgical efficiency compared to conventional fluoroscopic guidance. These findings suggest that the AIOOR system is a promising tool for improving the precision and efficiency of pedicle screw placement in spinal surgery.

## Data Availability

The data that support the findings of this study are available from the corresponding author upon reasonable request.

## Research registration unique identifying number (UIN)

The trial was registered in the Chinese Clinical Trial Registry (ChiCTR2000036039) (https://www.chictr.org.cn/showproj.html?proj=59052).
